# Investigating the association between the food inflammation scores of individuals and stroke in adults: an extreme gradient boosting machine learning model interpreted with shapley additive explanations

**DOI:** 10.1186/s41043-026-01342-6

**Published:** 2026-05-18

**Authors:** Zhiwen Yan, Kang Luo, Qinghuan Yang, Xiaoqing Liu, Huan Zhao, Yuan Gao, Qiang Zhang, Jun Mu

**Affiliations:** 1 Department of Neurology, The First Affiliated Hospital of Chongqing Medical University, Chongqing, 400016 China; 2 Department of Geriatrics, The First Affiliated Hospital of Chongqing Medical University, Chongqing, 400016 China; 3 College of Life Sciences, Institute of Biomedical Engineering, Qingdao University, Qingdao, 266071 Shandong China; 4 Department of Rheumatology and Immunology, The Second Affiliated Hospital of Zhejiang Chinese Medical University, 310005 Hangzhou, China

**Keywords:** Food Inflammation Index, Stroke, NHANES, Machine Learning, Dietary Patterns, Nutrition

## Abstract

**Background:**

Chronic systemic inflammation is a pivotal modifiable risk factor for stroke. The food-based Food Inflammation Index (FII) offers a novel approach to assess dietary inflammatory potential, yet the association between its derivative, the Food Inflammation Scores of Individuals (FISI), and stroke prevalence remains to be elucidated.

**Methods:**

This study analyzed a cohort of 19,681 adults from the NHANES (2007–2018) database. The FISI-stroke association was assessed using multivariable logistic regression and machine learning models (XGBoost), interpreted via SHAP analysis.

**Results:**

Higher FISI scores were positively associated with increased stroke prevalence in a dose-dependent manner. Specifically, a one-unit rise in FISI34, FISI26-USDA, and FISI26-CHINA corresponded to 7%, 18%, and 22% higher stroke odds, respectively. XGBoost modeling identified FISI34 as a key predictor, corroborating regression findings.

**Conclusions:**

This study establishes a robust link between higher FISI, derived from the FII, and stroke risk. The FII framework surpasses nutrient-based indices by providing personalized, actionable, food-specific guidance for stroke prevention through anti-inflammatory diets.

**Graphical Abstract:**

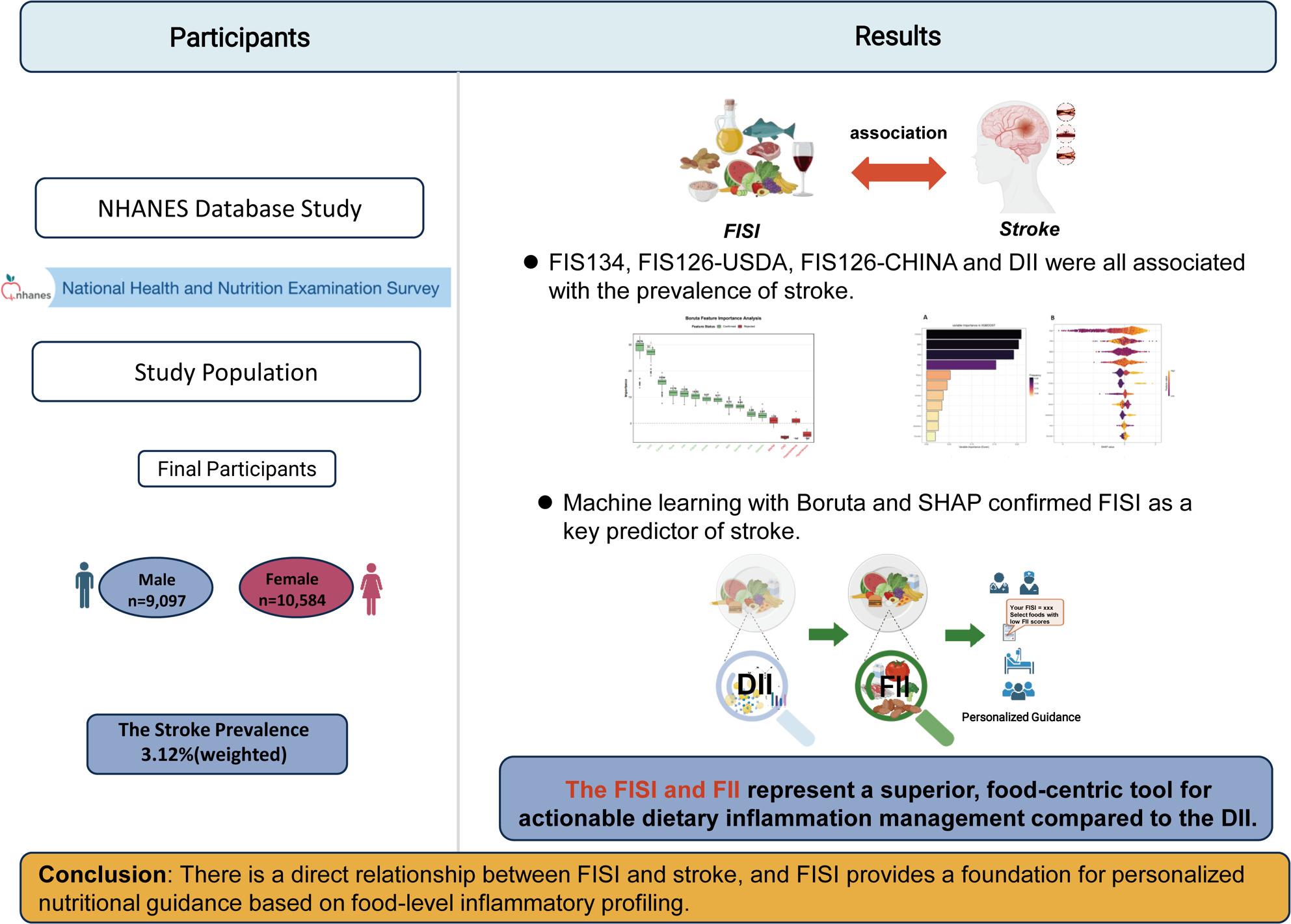

**Supplementary Information:**

The online version contains supplementary material available at 10.1186/s41043-026-01342-6.

## Introduction

Stroke **remains a leading cause of global morbidity and mortality**, with profound implications for public health [1]. The 2019 Global Burden of Disease Study revealed marked rises in stroke burden over recent decades, including a 70% increase in incidence, 43% in mortality, 85% in prevalence, and 32% in disability-adjusted life years (DALYs) [2, 3]. In addition to high fatality rates, stroke often leaves survivors with chronic disabilities, placing heavy economic and emotional burdens on patients, caregivers, and healthcare systems [1, 4].

The pathogenesis of stroke is multifactorial, encompassing genetic predisposition, unfavorable lifestyle factors, environmental influences, and comorbid chronic conditions [5, 6]. Emerging evidence over the past decade has established persistent low-grade systemic inflammation as an important independent risk factor for stroke [7, 8]. Accumulating research indicates that sustained inflammation accelerates cerebrovascular events through multiple pathways, including endothelial dysfunction, acceleration of atherogenesis, and heightened thrombotic tendency [9, 10]. Furthermore, research has indicated that non-dietary factors, such as functional dependence, are significantly associated with increased cardiovascular risk, adding complexity to the prevention of cerebrovascular events [11].

Dietary patterns are among the most influential modifiable determinants of systemic inflammatory status, and consequently, stroke risk. Compelling evidence has consistently linked specific eating habits with stroke occurrence [12]. Notably, Western-style diets dominated by high intakes of saturated fats, refined carbohydrates, and caloric excess promote sustained low-grade inflammation and significantly heighten the likelihood of cerebrovascular events [13]. Conversely, adherence to the Mediterranean dietary pattern-characterized by abundant fruits, vegetables, legumes, nuts, whole grains, and extra-virgin olive oil-has been repeatedly associated with reduced inflammatory markers and improved cerebrovascular protection [14–17]. These observations underscore the considerable preventive potential of tailored nutritional strategies in lowering stroke burden.

The FII [18] was developed as an advancement of the DII [19], incorporating food-specific inflammation scores, daily reference nutrient values (NRVs), and an expanded food composition database. This refinement allows FII to provide a precise quantification of the inflammatory potential of individual foods, making it a promising and valuable tool for personalized nutritional interventions and chronic disease prevention. The Food Inflammation Scores of Individuals (FISI), including FISI34, FISI26-USDA, and FISI26-CHINA, were developed from the original Food Inflammation Index (FII) to more precisely quantify the pro- or anti-inflammatory potential of a person’s habitual diet. Among them, FISI34 is calculated based on 34 key nutrients, providing a comprehensive assessment. Both FISI34 and FISI26-USDA rely on nutrient composition data from the United States Department of Agriculture (USDA) database together with U.S. Dietary Reference Intakes, whereas FISI26-CHINA uses the same methodology but applies nutrient values and reference standards from the Chinese Dietary Reference Intakes (NRVs).

Earlier research has shown that elevated DII scores correlate with higher inflammation levels and a greater occurrence of stroke and other chronic illnesses, while lower DII scores suggest anti-inflammatory dietary characteristics [20–23]. Although the DII remains the most widely adopted tool for characterizing diet-related inflammation, the FII and its subsequent individualized indices demonstrate clear advantages in the context of precision and personalized nutrition. However, research on the association between FISI34, FISI26-USDA, FISI26-CHINA, and stroke prevalence is currently lacking, and systematic investigations into their relationship with stroke are yet to be conducted.

Using NHANES data spanning 2007–2018, the present study investigates the associations of three nutrient-based inflammatory scores —FISI34, FISI26-USDA, and FISI26-CHINA— with the prevalence of stroke in adults. Through comparative analysis of these individualized dietary inflammation indices, we aim to characterize the contribution of diet-driven inflammation to stroke risk and to inform the design of precision dietary interventions targeting specific macronutrients and food groups.

## Methods

### Study layout and participant details

The present study utilized publicly available data from six NHANES cycles (2007–2018). All participants provided written informed consent, and study protocols were approved by the NCHS Research Ethics Review Board. Data were collected through household interviews, standardized physical examinations, laboratory testing at mobile examination centers, and 24-hour dietary recalls [24].

### Study population

**Fig. 1:**
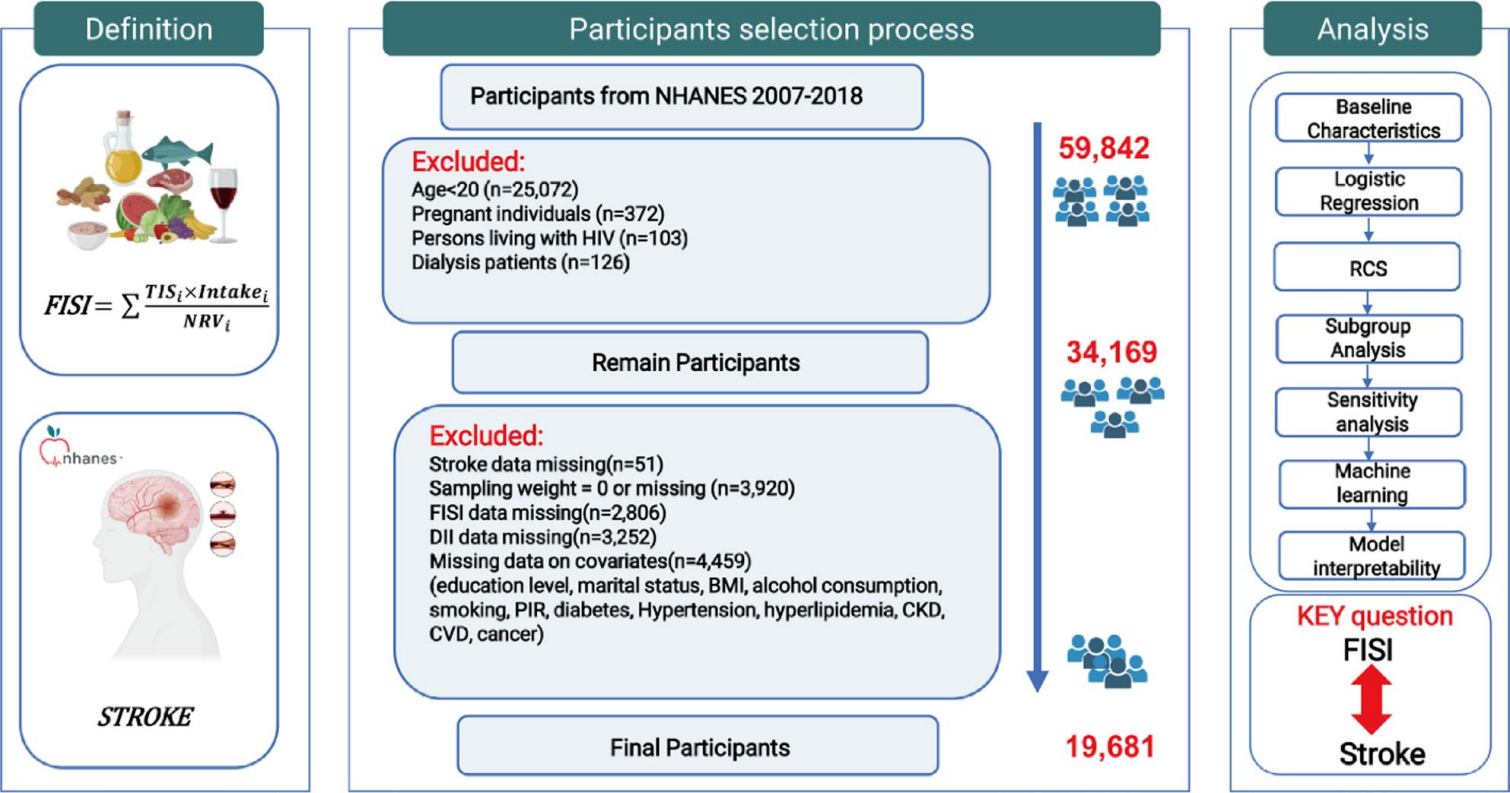
༎Participant selection flowchart

Participants were selected from six consecutive NHANES cycles (2007–2018 (initial *n* = 59,842). we excluded participants age < 20 years (*n* = 25,072), pregnancy (*n* = 372), known HIV infection (*n* = 103), receiving dialysis (*n* = 126), missing stroke outcome data (*n* = 51), absent or zero examination weights (*n* = 3,920), incomplete FISI variables (*n* = 2,806), incomplete DII variables (*n* = 3,252), and missing key covariates (*n* = 4,459). After these exclusions, 19,681 adults were retained for the final analytic sample (Fig. 1).

### Stroke ascertainment

Stroke status was determined by self-report during the household interview. Participants were asked: “Has a doctor, physician assistant, or other health professional ever told you that you had a stroke?” Those who answered “yes” were classified as having a history of stroke [25].

### Calculation of FISI34, FISI26-USDA, FISI26-CHINA and DII

The values of FISI34, FISI26-USDA, FISI26-CHINA and DII were calculated using the final retained analysis samples after the above screening (*n* = 19,681). The dietary data of the study participants are processed as follows: if complete dietary records for two days are available, the average intake is taken as the daily intake; if the second day’s data is missing, the first day’s data is used; for individuals with data for only one day, the data from that day is directly used. We acknowledge potential biases in 24-hour recalls, such as underreporting (e.g., participants may forget or minimize high-calorie intakes) and day-to-day variation (e.g., atypical eating days), which could affect accuracy. To address these limitations, we prioritized averaged two-day data where available and used the first day’s data for missing second-day records across different cycles.

The FISI was developed to estimate the cumulative inflammatory impact of daily dietary intake [18]. The methodology for FISI construction and parameter selection was based on our previously established and validated protocols [26]. The calculation follows the formula:

1 $$\:\boldsymbol{F}\boldsymbol{I}\boldsymbol{S}\boldsymbol{I}=\sum\:\frac{{\boldsymbol{T}\boldsymbol{I}\boldsymbol{S}}_{\boldsymbol{i}}\times\:{\boldsymbol{I}\boldsymbol{n}\boldsymbol{t}\boldsymbol{a}\boldsymbol{k}\boldsymbol{e}}_{\boldsymbol{i}}}{{\boldsymbol{N}\boldsymbol{R}\boldsymbol{V}}_{\boldsymbol{i}}}$$

Where:

$\:{Intake}_{i}$ is the individual’s average daily intake of the i-th nutrient.

$\:{TIS}_{i}$ (Total Inflammation Score) represents the specific inflammatory weight of each nutrient. These weights were derived from a comprehensive meta-analysis of the relationship between 39 dietary components and six established inflammatory biomarkers (IL-1β, IL-4, IL-6, IL-10, TNF-α, and CRP). A positive TIS_i_ indicates pro-inflammatory potential, while a negative value indicates anti-inflammatory effects.

$\:{NRV}_{i}$ (Nutrient Reference Value) is the reference intake used for normalization. For the US-based models (FISI34 and FISI26-USDA), $\:NR{V}_{i}$ values were set according to the U.S. Dietary Guidelines for Americans (DGA) 2020–2025 (standardized for ages 31–50 at 2200 kcal). For the Chinese model (FISI26-CHINA), $\:NR{V}_{i}$ was adjusted based on the 2023 Edition of Dietary Reference Intakes (DRIs) for China.

**Nutrient Selection and Indices Variants**: FISI34: This index was adapted from the original FISI39 framework. Due to the lack of data for five flavonoids (Flavan-3-ols, Flavanones, Flavones, Flavonols, and Isoflavones) in the NHANES 2011–2018 cycles, the nutrient count was adjusted to 34 key components with established inflammatory effects, as detailed in Supplementary Table S1^18^. FISI26-USDA and FISI26-CHINA use 26 core nutrients selected for their representativeness across USDA-FCT and China-FCT datasets, detailed in Supplementary Table S1, excluding non-core items like tea and onions. FISI26-USDA is built with USDA-FCT data and NRVs from DGA 2020–2025 to validate the 26 nutrients’ effectiveness in reflecting dietary inflammation in US contexts. FISI26-CHINA utilizes the same 26 nutrients, incorporating China-FCT data and NRVs from the 2023 Edition of Dietary Reference Intakes for China, customized for Chinese eating habits [27, 28]. The dual models assess NRVs’ impact and validate cross-cultural applicability, addressing food group heterogeneity.

**DII Calculation**: For the present investigation, DII scores were computed via the R software package *dietaryindex* (available at https://github.com/jamesjiadazhan/dietaryindex), following methodologies established in prior literature [19, 29]. Although the original DII was designed based on 45 dietary components, the DII based on these 28 components(Supplementary Table S2) is widely recognized as an effective tool for evaluating the inflammatory potential of diets, and its reliability and utility have been validated in multiple studies [30, 31], including those related to stroke [22, 32]. Detailed calculations are provided in the Supplementary Information S1.

### Covariates

Based on previous literature [22, 33–35], All models were adjusted for age, sex, race/ethnicity, poverty-to-income ratio education level, BMI, smoking status, alcohol consumption, and physician-diagnosed hypertension, diabetes, cardiovascular disease, chronic kidney disease, and hyperlipidemia. Age was used as a continuous variable and also categorized into two groups ($\:<60$ and $\:\ge\:60$ years). Sex was categorized as male or female. Race/ethnicity was classified into five groups: Non-Hispanic White, Non-Hispanic Black, Mexican American, Other Hispanic, and Other Race. Education level was divided into three categories: $\:<$ High school, Completed high school, and $\:>$High school. Economic status was assessed using the Poverty-to-Income Ratio (PIR), which was categorized into three levels: $\:<1.3$ (low income), $\:1.3\mathrm{\--}3.5$ (middle income), and $\:\ge\:3.5$ (high income) [36, 37], Body Mass Index (BMI) was calculated as weight in kilograms divided by the square of height in meters ($\:kg/{m}^{2}$) and categorized as $\:<25$, $\:25-30$, and $\:\ge\:30$ $\:kg/{m}^{2}$. Smoking status was classified as: never smokers ($\:<100$ cigarettes in life), former smokers ($\:\ge\:100$ cigarettes but quit at the time of interview), and current smokers ($\:\ge\:100$ cigarettes and still smoking) [38]. Alcohol consumption was categorized based on drinking frequency and volume into: lifetime abstainers, former drinkers, current moderate drinkers, and current heavy drinkers [39]. Health-related covariates included hypertension, diabetes, cardiovascular disease (CVD), chronic kidney disease (CKD), and hyperlipidemia. These were determined based on self-reported history of diagnosis by a healthcare professional or through specific laboratory criteria (e.g., hemoglobin A1c $\:\ge\:6.5\mathrm{\%}$ for diabetes, or specific lipid levels for hyperlipidemia) [40, 41]. These conditions were defined using a combination of self-reported medical history, medication use, and standardized clinical and laboratory measurements. Specifically, hypertension was identified by a self-reported physician diagnosis, current use of antihypertensive medication, a systolic blood pressure (SBP) ≥140 mmHg, or a diastolic blood pressure (DBP) ≥90 mmHg. Diabetes was ascertained via a self-reported diagnosis, hemoglobin A1c (HbA1c) ≥6.5%, fasting plasma glucose (FPG) ≥126 mg/dL, or a 2-hour post-load plasma glucose ≥200 mg/dL during an oral glucose tolerance test (OGTT)[38, 39]. CKD was defined according to the 2021 KDIGO clinical practice guidelines, indicated by a urinary albumin-to-creatinine ratio (ACR) ≥30 mg/g or an estimated glomerular filtration rate (eGFR) <60 mL/min/1.73 m². Finally, hyperlipidemia (dyslipidemia) was determined by the use of cholesterol-lowering medications or specific lipid profile abnormalities, including serum total cholesterol (TC) ≥200 mg/dL, triglycerides ≥150 mg/dL, low-density lipoprotein (LDL) ≥130 mg/dL, or high-density lipoprotein (HDL) <40 mg/dL in men and <50 mg/dL in women.

### Statistical analysis

All analyses were performed using survey-weighted methods that accounted for NHANES complex sampling design. For the integrated 2007–2018 dataset, multi-year sample weights were calculated by dividing the day 1 dietary intake weights (WTDRD1) by six, following NCHS analytic recommendations. Statistical procedures were conducted in R (version 4.5.0) using the survey package. Weighted baseline characteristics were compared using design-adjusted Wald tests for continuous variables and Rao-Scott adjusted $\:{\chi\:}^{2}$ tests for categorical variables. The associations of FISI34, FISI26-USDA, FISI26-CHINA, and DII with prevalent stroke were examined using survey-weighted multivariable logistic regression. Each inflammatory score was first modeled as a continuous variable and subsequently categorized into quartiles (Q1-Q4, with Q1 serving as the reference). P-for-trend was calculated by treating the median value of each quartile as a continuous variable. Three sequential models were constructed: Model 1, Model 2, and Model 3. Non-linear dose-response relationships were characterized using restricted cubic splines (RCS) with 3 knots (10th, 50th, and 90th percentiles).

For feature selection, the Boruta algorithm was implemented with 100 iterations (maxRuns = 100) and a significance threshold (pValue) of 0.01. To optimize the machine learning models (XGBoost and LGBM), we employed a grid search strategy for hyperparameter tuning combined with 10-fold cross-validation to mitigate the risk of overfitting. Specifically, for the XGBoost model, we conducted a grid search over key hyperparameters, including: learning rate (0.01, 0.05, 0.1), maximum depth of trees (3, 6, 9), subsample ratio (0.6, 0.8, 1.0), and number of estimators (100, 500, 1000). The optimal configuration was determined based on the highest Area Under the Curve (AUC) achieved through the 10-fold cross-validation process. Model interpretability was further explored using SHAP (SHapley Additive exPlanations) values. Sensitivity analysis was conducted using Propensity Score Matching (PSM) to ensure the robustness of our findings. A 1:1 nearest-neighbor matching algorithm was applied with a caliper (matching tolerance) of 0.2 to balance covariates including age, sex, race, education, marital status, smoking, alcohol use, BMI, and comorbidities. Post-matching balance was evaluated using standardized mean differences (SMD) and love plots. A two-tailed *P* < 0.05 was considered statistically significant.

## Results

### Overview of the NHANES demographic

In Table 1, the baseline characteristics of 19,681 participants, divided into non-stroke (*n* = 18,883) and stroke (*n* = 798) groups. The crude prevalence of stroke, calculated as the proportion of affected individuals (798/19,681), was 4.05%. The weighted prevalence, derived by dividing the sum of weights for individuals with stroke (4,648,352) by the total sum of weights (148,853,819), was 3.12% in the US population. The average age was 48.9 years, with a balanced gender distribution (45% male, 55% female). There were notable differences (*p* < 0.05) among groups regarding age, race, education level, PIR, BMI, alcohol consumption, diabetes, hypertension, hyperlipidemia, CVD, CKD, cancer, FISI levels. There were no notable differences in terms of gender or marital status.

**Table 1:** Baseline characteristics of participants with stroke

Characteristic	Total (*n* = 19681)	Non-stroke (*n* = 18883)	Stroke (*n* = 798)	*P* value
**Age**,** years**	48.9 (17.0)	48.3 (16.8)	65.3 (13.3)	**< 0.001**
**Age group**,** n (%)**				**< 0.001**
< 60	12,548 (70.7%)	12,354 (72.1%)	194 (29.6%)	
≥ 60	7,133 (29.3%)	6,529 (27.9%)	604 (70.4%)	
**Gender**,** n (%)**				0.13
Female	10,584 (54.7%)	10,162 (54.6%)	422 (58.5%)	
Male	9,097 (45.3%)	8,721 (45.4%)	376 (41.5%)	
**Race**,** n (%)**				**< 0.001**
Non-Hispanic White	8,787 (69.0%)	8,366 (68.9%)	421 (71.4%)	
Non-Hispanic Black	4,012 (10.3%)	3,799 (10.2%)	213 (14.1%)	
Mexican American	2,819 (8.0%)	2,757 (8.1%)	62 (4.3%)	
Other Race	2,094 (7.4%)	2,042 (7.4%)	52 (7.3%)	
Other Hispanic	1,969 (5.3%)	1,919 (5.4%)	50 (2.9%)	
**Education level**,** n (%)**				**< 0.001**
< High school	1,708 (4.3%)	1,603 (4.2%)	105 (8.6%)	
> High school	15,423 (86.1%)	14,874 (86.5%)	549 (75.9%)	
Completed high school	2,550 (9.6%)	2,406 (9.4%)	144 (15.5%)	
**Marital status**,** n (%)**				**0.04**
Married/Living with partner	11,918 (63.7%)	11,492 (63.9%)	426 (58.8%)	
Widowed/Divorced/Separated /Never married	7,763 (36.3%)	7,391 (36.1%)	372 (41.2%)	
**PIR**	3.02 (1.65)	3.04 (1.65)	2.40 (1.50)	**< 0.001**
**PIR group**,** n (%)**				**< 0.001**
< 1.3	5,978 (21.0%)	5,676 (20.8%)	302 (29.5%)	
1.3–3.5	6,103 (43.4%)	5,962 (44.0%)	141 (24.8%)	
≥ 3.5	7,600 (35.6%)	7,245 (35.3%)	355 (45.8%)	
**BMI**,** kg/m²**	29.3 (6.9)	29.3 (6.9)	30.5 (7.3)	**0.001**
**BMI group**				**< 0.001**
< 25 kg/m²	5,323 (28.3%)	5,143 (28.4%)	180 (23.6%)	
25–30 kg/m²	7,965 (39.3%)	7,592 (39.0%)	373 (49.0%)	
≥ 30 kg/m²	6,393 (32.4%)	6,148 (32.6%)	245 (27.3%)	
**Alcohol consumption**,** n (%)**				**< 0.001**
Lifetime abstainers	2,779 (11.0%)	2,666 (10.9%)	113 (13.0%)	
Former drinkers	5,128 (21.8%)	4,765 (21.2%)	363 (39.8%)	
Current moderate drinkers	6,054 (34.8%)	5,847 (35.0%)	207 (30.4%)	
Current heavy drinkers	5,720 (32.4%)	5,605 (32.9%)	115 (16.8%)	
**Smoking status**,** n (%)**				**< 0.001**
Never smoker	11,237 (57.7%)	10,924 (58.3%)	313 (40.1%)	
Former smoker	4,972 (25.5%)	4,668 (25.1%)	304 (38.3%)	
Current smoker	3,472 (16.8%)	3,291 (16.6%)	181 (21.7%)	
**Comorbidities**,** n (%)**				
Diabetes	3,941 (15.2%)	3,597 (14.4%)	344 (39.0%)	**< 0.001**
Hypertension	8,698 (38.9%)	8,047 (37.7%)	651 (76.7%)	**< 0.001**
Cancer	2,083 (11.2%)	1,894 (10.8%)	189 (23.4%)	**< 0.001**
Hyperlipidemia	14,373 (72.2%)	13,672 (71.8%)	701 (87.1%)	**< 0.001**
CKD	3,728 (15.5%)	3,354 (14.6%)	374 (41.7%)	**< 0.001**
CVD	1,741 (7.1%)	1,448 (6.1%)	293 (35.5%)	**< 0.001**
**FISI34**	−6.14 (2.67)	−6.16 (2.67)	−5.49 (2.72)	**< 0.001**
**FISI26-CHINA**	−2.54 (1.08)	−2.55 (1.08)	−2.20 (1.01)	**< 0.001**
**FISI26-USDA**	−3.04 (1.23)	−3.05 (1.23)	−2.66 (1.20)	**< 0.001**

DII: dietary inflammatory index; BMI: body mass index; PIR: poverty income ratio CVD: cardiovascular disease; CKD: chronic kidney disease

FISI34: Food inflammation scores of individuals based on 34 nutrient components;

FISI26-USDA: food inflammation scores of individuals based on 26 nutrient components, using USDA food composition (details in Supplementary Table S1)

FISI26-CHINA: food inflammation scores of individuals based on 26 food and nutrient components, using Chinese food composition (details in Supplementary Table S1)

mean ± SD for continuous; n (%) for categorical; *P* < 0.05 indicates a significant difference

### Association between FISI34, FISI26-USDA, FISI26-CHINA, DII and stroke

The association between various Food Inflammation Scores (FISI34, FISI26-USDA, FISI26-CHINA, and DII) and stroke evaluation was conducted with three different models: Model 1, Model 2, and Model 3. Table 2 shows that in every model, all scores had a positive correlation with stroke, with odds ratios (ORs) between 1.07 and 1.22 in Model 3(p values are all less than 0.05). Higher quartiles consistently showed stronger associations with stroke compared to Q1, with significant trends.

**Table 2:** The relationship between FISI34, FISI26-USDA, FISI26-CHINA, DII, and stroke

Variables	Model1			Model2			Model3		
	OR	95% CI	*P* value	OR	95% CI	*P* value	OR	95% CI	*P* value
FISI34	1.11	1.06, 1.17	**< 0.001**	1.1	1.04, 1.17	**< 0.001**	1.07	1.02, 1.13	**0.005**
FISI34 quantiles									
Q1	—	—		—	—		—	—	
Q2	1.06	0.76, 1.48	0.7	1.01	0.72, 1.43	0.9	1.01	0.72, 1.42	0.9
Q3	1.51	1.12, 2.03	**0.008**	1.42	1.04, 1.95	**0.03**	1.36	0.99, 1.87	0.056
Q4	2	1.51, 2.65	**< 0.001**	1.94	1.41, 2.67	**< 0.001**	1.63	1.19, 2.23	**0.003**
P for trend			**< 0.001**			**< 0.001**			**0.001**
FISI26-USDA	1.33	1.21, 1.47	**< 0.001**	1.32	1.19, 1.47	**< 0.001**	1.18	1.06, 1.30	**0.002**
FISI26-USDA quantiles									
Q1	—	—		—	—		—	—	
Q2	1.82	1.32, 2.51	**< 0.001**	1.72	1.24, 2.39	**0.001**	1.59	1.13, 2.22	**0.008**
Q3	2.02	1.44, 2.84	**< 0.001**	1.95	1.38, 2.77	**< 0.001**	1.62	1.13, 2.33	**0.01**
Q4	2.89	2.16, 3.88	**< 0.001**	2.72	2.02, 3.67	**< 0.001**	1.99	1.46, 2.70	**< 0.001**
P for trend			**< 0.001**			**< 0.001**			**0.001**
FISI26-CHINA	1.4	1.25, 1.56	**< 0.001**	1.41	1.25, 1.59	**< 0.001**	1.22	1.09, 1.38	**< 0.001**
FISI26-CHINA quantiles									
Q1	—	—		—	—		—	—	
Q2	1.55	1.18, 2.04	**0.002**	1.51	1.14, 2.02	**0.005**	1.39	1.04, 1.86	**0.029**
Q3	1.91	1.38, 2.64	**< 0.001**	1.87	1.32, 2.63	**< 0.001**	1.55	1.08, 2.21	**0.017**
Q4	2.47	1.91, 3.21	**< 0.001**	2.43	1.86, 3.18	**< 0.001**	1.73	1.32, 2.27	**< 0.001**
P for trend			**< 0.001**			**< 0.001**			**0.001**
DII	1.25	1.18, 1.34	**< 0.001**	1.26	1.18, 1.34	**< 0.001**	1.15	1.07, 1.23	**< 0.001**
DII quantiles									
Q1	—	—		—	—		—	—	
Q2	1.49	1.07, 2.06	**0.018**	1.56	1.11, 2.18	**0.011**	1.4	0.98, 1.98	0.063
Q3	1.68	1.21, 2.34	**0.003**	1.77	1.26, 2.50	**0.001**	1.41	0.98, 2.03	0.067
Q4	2.64	1.98, 3.53	**< 0.001**	2.65	1.98, 3.55	**< 0.001**	1.82	1.35, 2.48	**< 0.001**
P for trend			**< 0.001**			**< 0.001**			**0.001**

Model 1: Unadjusted

Model 2: Adjusted for age, gender, and race

Model 3: Adjusted for age, gender, race, marital status, education level, PIR, BMI, smoking status, alcohol consumption, cancer, hypertension, diabetes, hyperlipidemia, CVD, and CKD

Note: OR: Odds Ratio, CI: Confidence Interval; Q: Quartiles; PIR: poverty-to-income ratio; BMI: body mass index; CVD: cardiovascular disease; CKD: chronic kidney disease; DII: dietary inflammatory index; FISI34: Food inflammation scores of individuals based on 34 nutrient components; FISI26-USDA: food inflammation scores of individuals based on 26 nutrient components, using USDA food composition (details in Supplementary Table S1); FISI26-CHINA: food inflammation scores of individuals based on 26 food and nutrient components, using Chinese food composition (details in Supplementary Table S1)

*P* < 0.05 indicates a significant difference

### Nonlinear relationship analysis

**Fig. 2:**
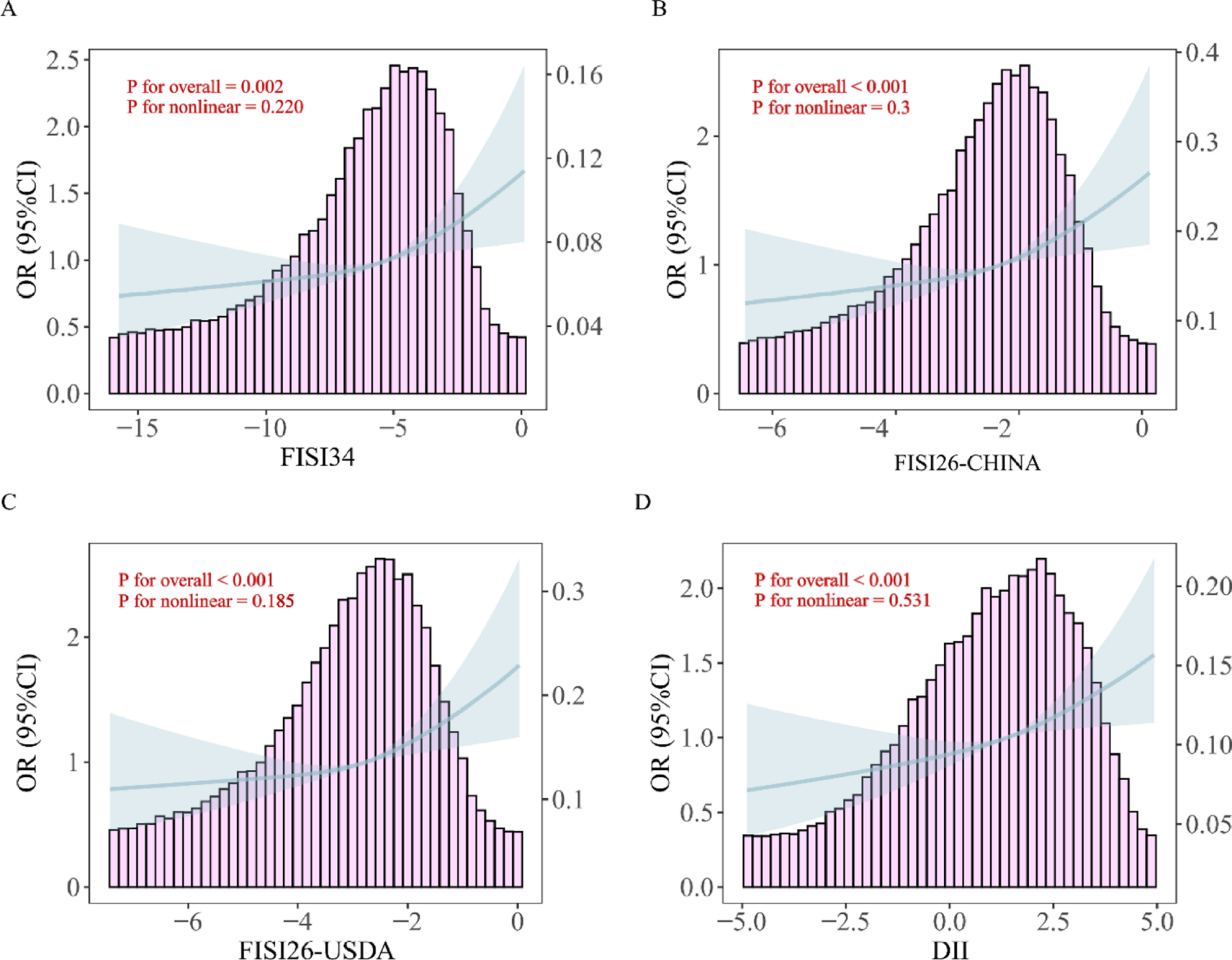
Nonlinear and Linear relationship between FISI34, FISI26-USDA, FISI26-CHINA, DII, and stroke

Figure 2 illustrates restricted cubic splines that depict the dose-response relationship between FISI34, FISI26-USDA, FISI26-CHINA, DII, and stroke. Each plot highlights linear trends: Figure 2A, B and C, and D show a linear relationship between FISI34, FISI26-USDA, FISI26-CHINA, DII, and stroke, with the prevalence of stroke gradually increasing as the FISI values rise.

### Subgroup analysis

**Fig. 3:**
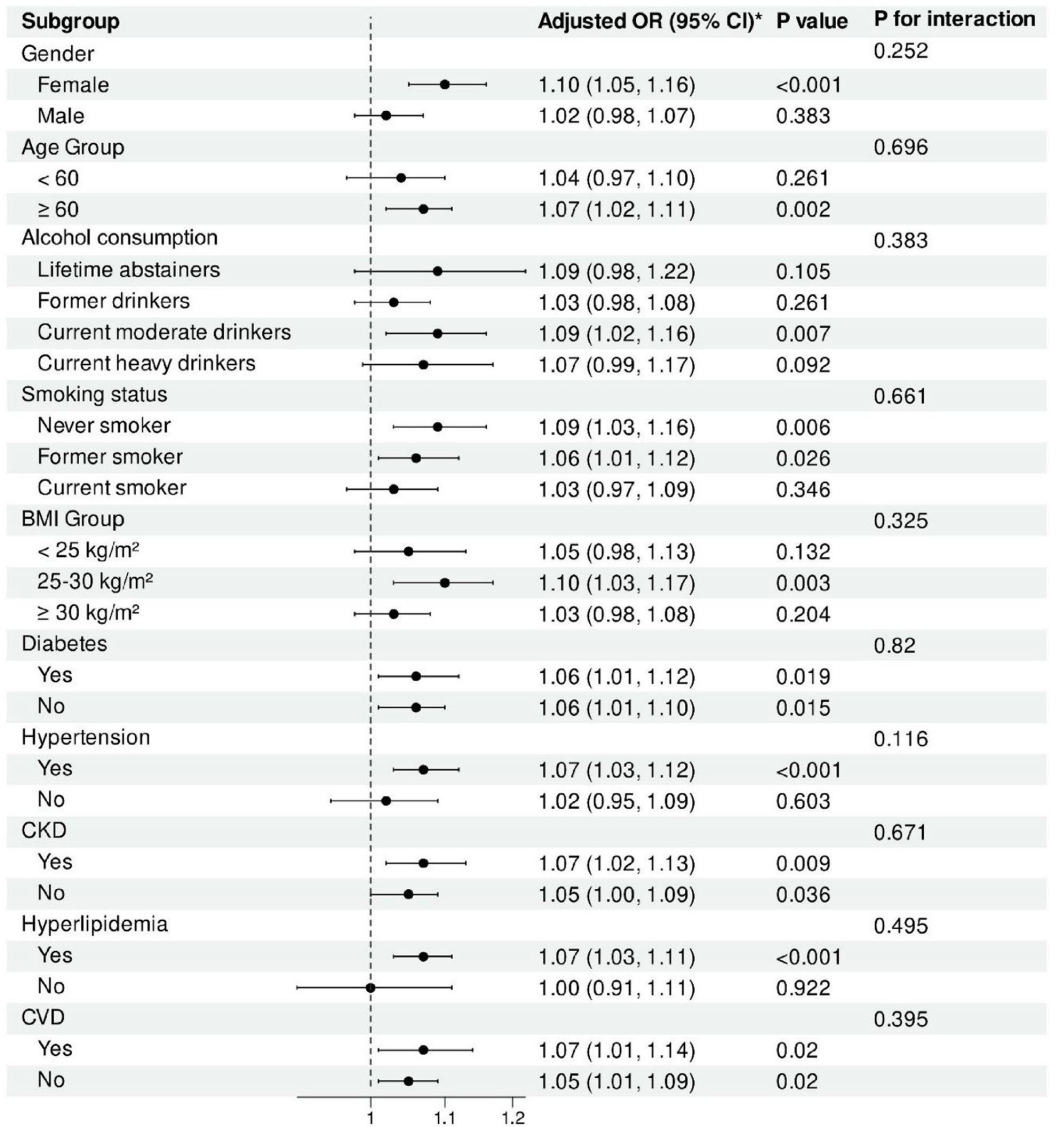
Examination of the relationship between FISI34 and stroke across different subgroups. Taking into account age, gender, race, marital status, education level, PIR, BMI, smoking habits, alcohol use, hypertension, diabetes, cardiovascular disease, chronic kidney disease, and cancer

To evaluate the dependability of the connection between FISI34, FISI26-USDA, FISI26-CHINA and stroke, Subgroup analysis was carried out based on variables like age, gender, race, education level, PIR, BMI, smoking status, alcohol intake, cancer, hypertension, diabetes, hyperlipidemia, CVD, and CKD (Fig. 3 and Supplementary Figures S1 and S2). The results consistently indicated a positive link between them and stroke in all subgroups. There were no notable interactions found across subgroups (interaction p-value > 0.05).

### Identification of key variables for stroke using the Boruta Algorithm

**Fig. 4:**
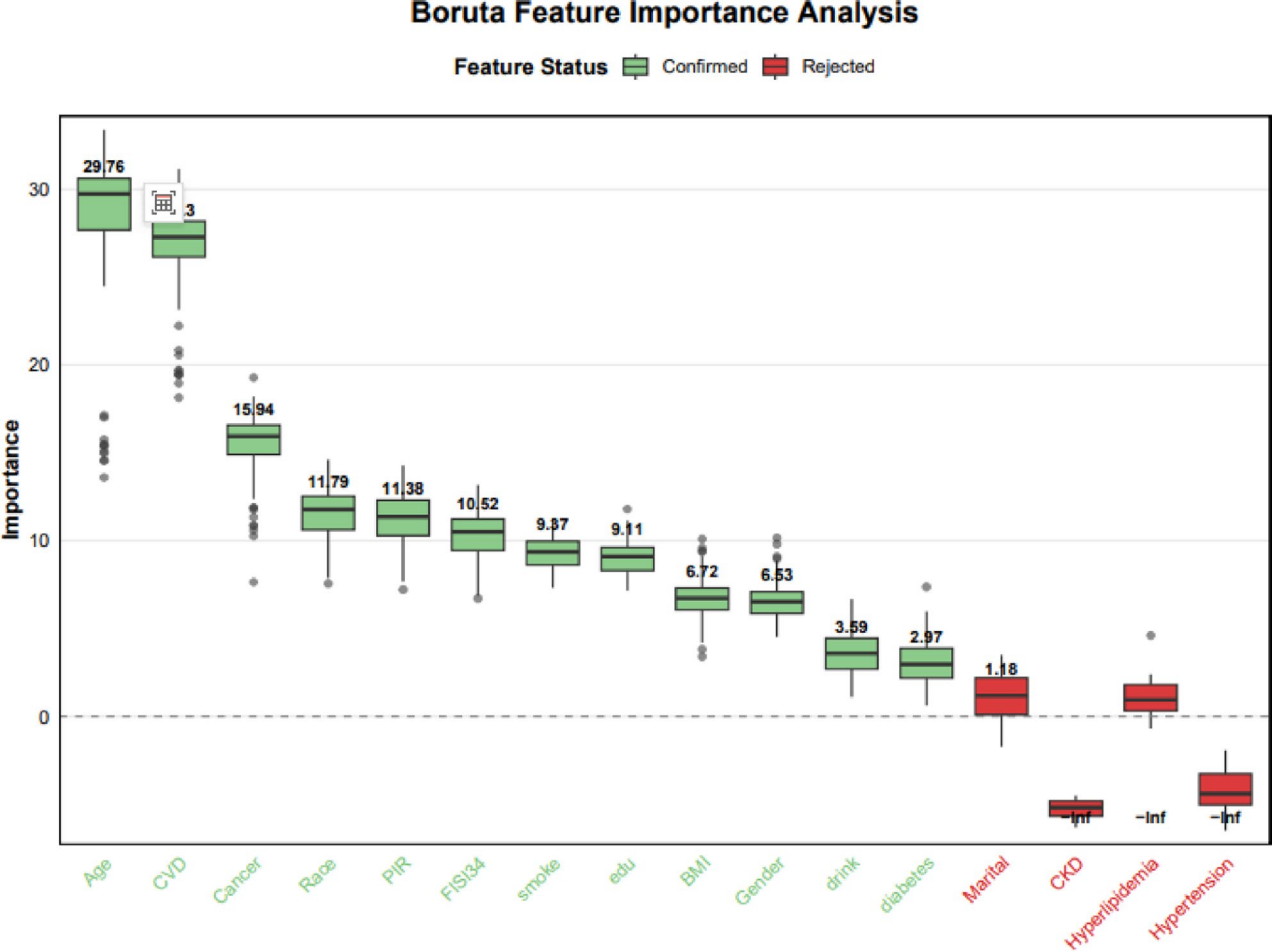
Ranking of critical stroke factors using the Boruta algorithm

Variables identified in the green zone, including FISI34, were validated as important factors in the Boruta algorithm analysis, highlighting their essential role in stroke. FISI34, in particular, was identified as a major factor, with its increased Z-score highlighting its considerable influence on the overall profile. Conversely, such as marital status, CKD, hypertension, diabetes, and hyperlipidemia, were classified as less influential in this model, although they showed significant associations with stroke in baseline analyses (Table 1). Refer to Fig. 4 for additional details.

### Assessing the performance of several machine learning models with the dataset

**Fig. 5:**
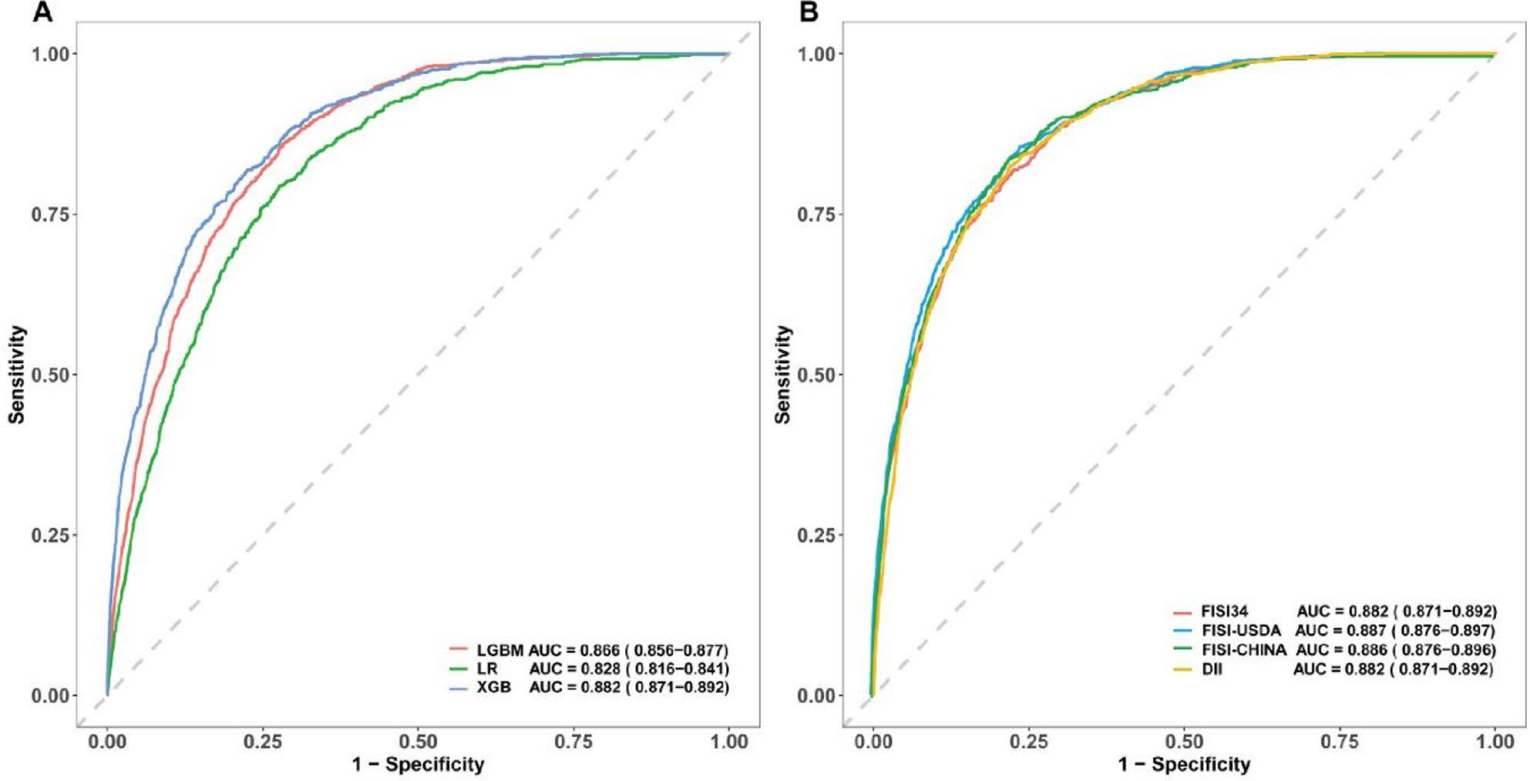
Comparison of Performance between Traditional Models and Machine Learning Models. Comparison of ROC between logistic regression model and two machine learning models (**A**). Comparison of ROC between four dietary indices in the fully adjusted model (**B**). XGB stands for extreme gradient boosting, AUC refers to the area under the curve, and CI means confidence interval

Three models were trained and assessed: Logistic Regression (LR), eXtreme Gradient Boosting (XGB), and Light Gradient Boosting Machine (LGBM)—using the full sample dataset, with performance metrics summarized in (Table 3; Fig. 5). According to the results, the XGB model reached the highest level of discrimination performance, achieving an AUC of 0.882 (95%CI: 0.871–0.892), along with the highest F1-score (0.229), specificity (0.775), and sensitivity (0.820), outperforming both LR and LGBM. Furthermore, we evaluated the predictive performance of FISI34, FISI26-USDA, FISI26-CHINA, and DII within the XGB model, which showed comparable capability in predicting stroke risk.

**Table 3:** Evaluation of the performance of several machine learning models using the dataset

Model	AUC 95% CI	F1 score	Precision	Recall	Neg Pred value	Pos Pred value	Specificity	Sensitivity
LR	0.828(0.816–0.841)	0.191	0.108	0.794	0.988	0.108	0.723	0.794
XGB	0.882(0.871–0.892)	0.229	0.133	0.820	0.990	0.133	0.775	0.820
LGBM	0.866(0.856–0.877)	0.225	0.131	0.806	0.990	0.131	0.774	0.806

LR stands for Logistic Regression, LGBM refers to Light Gradient Boosting Machine, XGB is Extreme Gradient Boosting, Neg Pred value means Negative Predicted Value, Pos Pred value indicates Positive Predictive Value, AUC represents Area Under the Curve, and CI denotes Confidence Interval

### Explaining the XGBoost model using SHAP

To elucidate the classification model and systematically investigate the association between FISI34 and stroke status, this study constructed a stroke status discrimination model based on cross-sectional data using XGBoost algorithm, together with SHapley Additive exPlanations (SHAP), an XAI approach, to elucidate the model’s outcomes.

**Fig. 6:**
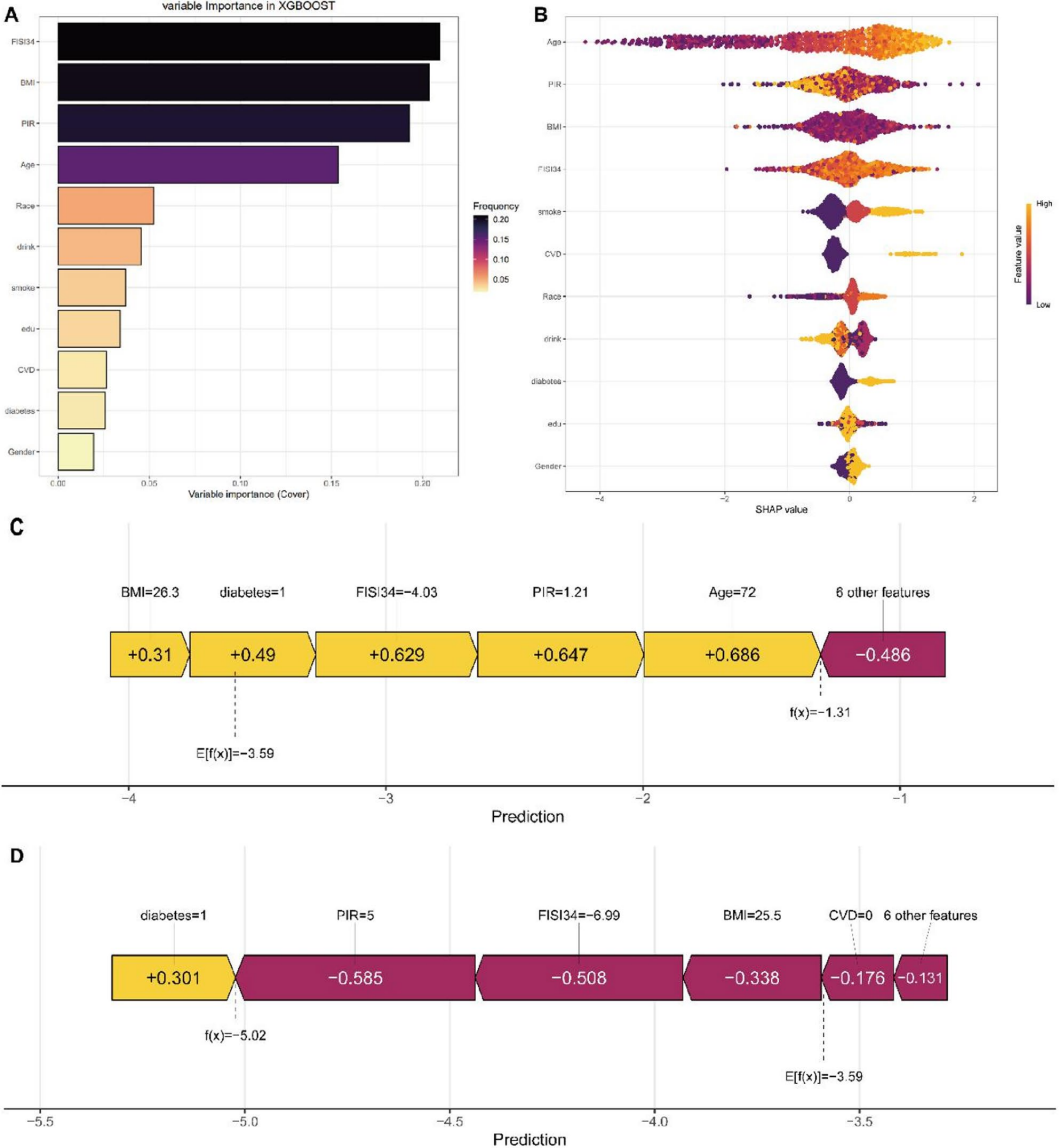
SHAP and individual case of XGB model. Feature importance ranking based on XGBoost model **(A)**, Beeswarm plot of SHAP values **(B)**, illustrating the distribution and impact direction (positive/negative) of each feature across all samples. The color represents the feature value from low (purple) to high (yellow-orange). Force plots explaining individual predictions **(C and D)**. Features pushing the prediction higher than the baseline are shown in yellow, while those decreasing the prediction are shown in magenta

According to Fig. 6A, the XGBoost model’s feature importance ranking revealed that FISI34 was the most crucial variable among those assessed, with a greater contribution than traditionally recognized associated factors such as BMI. This suggests that FISI34 has the highest relative influence in discriminating individual stroke status.

The overall direction and magnitude of this association were further clarified through the SHAP summary plot (Fig. 6B). The beeswarm plot displays the distribution of SHAP values for each feature. For FISI34, a clear pattern emerged: predominantly positive SHAP values were observed. This indicates that higher values of FISI34 are consistently associated with an increase in the model output value, corresponding to a greater likelihood of being classified into the stroke status.

Furthermore, interpretations from two representative individual cases corroborated these findings. In Fig. 6C, a higher value of FISI34 (FISI34 = −4.03, SHAP = + 0.629) acted as a major driver pushing the model’s prediction toward stroke status. Conversely, in Fig. 6D, a lower value of FISI34 (FISI34 = −6.99, SHAP = −0.508 contributed to reducing the probability of being classified as having stroke status.

### Sensitivity analysis

We performed a propensity score matching (PSM) analysis to further confirm the strength of the link between dietary inflammatory indices and stroke. Supplementary Table S3 shows that, after PSM (*N* = 798/group), the standardized mean differences for all matching variables (age, gender, education, marital, alcohol consumption, smoking, BMI, CKD, hypertension, hyperlipidemia, diabetes) were significantly reduced, with p-values all greater than 0.29. Multivariable logistic regression (Model 3), adjusting for multiple confounders, revealed significant associations with stroke for FISI34, FISI26-USDA, FISI26-CHINA, and DII (Supplementary Table S4). These findings confirm the robust positive association between dietary inflammatory indices and stroke, consistent with the primary analysis results.

## Discussion

### Dietary inflammation and stroke risk

This research was the pioneering effort to thoroughly examine the links between FISI34, FISI26-USDA, and FISI26-CHINA with stroke prevalence, and assessed the robustness of these associations across multiple adjustment levels. The findings showed that, even after accounting for different confounding variables, all three FISI scores had a significant positive correlation with stroke prevalence, with a noticeable dose-dependent trend as FISI scores rose. Analyses using restricted cubic splines showed linear connections between FISI scores and the prevalence of stroke. Crucially, FISI26-USDA and FISI26-CHINA, despite using fewer nutrients (26 vs. 34 in FISI34), maintained strong associations with stroke, suggesting that streamlined dietary inflammatory indices can effectively capture diet-related stroke risk. Earlier research has repeatedly demonstrated a link between DII and stroke [22, 32, 42], highlighting the influence of dietary inflammation on stroke. Furthermore, our findings align with the broader understanding of stroke as a multifactorial condition. Beyond dietary inflammation, other non-dietary contributors, such as functional dependence, have been shown to play a critical role in long-term cardiovascular health among middle-aged and older populations [11]. These collective insights emphasize the necessity of comprehensive intervention strategies for stroke prevention.

### Comparative advantages: FISI vs. DII

**Fig. 7:**
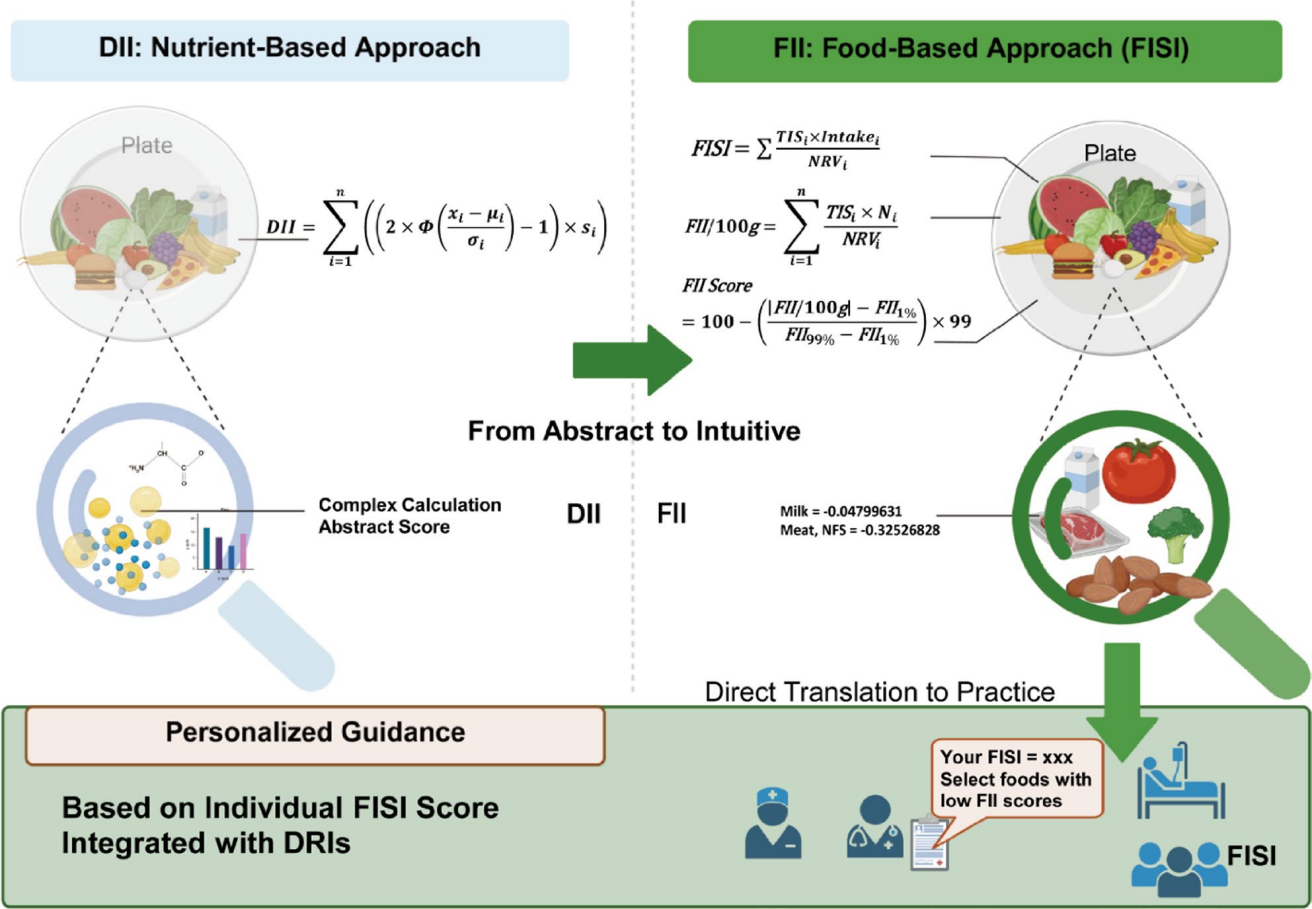
From Nutrients to Foods: The Evolution of Dietary Inflammation Assessment from the Abstract DII to the Clinically Actionable FII. (Left Panel) The DII is based on the abstract calculation of nutrient intakes (e.g., specific fatty acids, vitamins), which requires complex translation into dietary patterns and lacks immediate intuitiveness for clinical practice. (Right Panel) In contrast, the FII evaluates the inflammatory potential of whole foods directly (e.g., salmon, broccoli, tomatoes), with each food assigned a specific FII score. This food-based approach is intuitive and easily understood by both healthcare professionals and consumers. (Bottom Panel) The individual Food Inflammation Score (FISI), derived from the FII framework and integrated with Dietary Reference Intakes (DRIs), directly generates personalized, actionable guidance (e.g., specific food substitution advice: “Replace two servings of red meat with fish per week”). This enables clinicians to provide precise, evidence-based dietary recommendations. The right-pointing arrow signifies the evolution from the abstract DII to the intuitive FII. The downward-pointing arrow demonstrates the direct translation of the FII into practical application

Emphasizing plant-based foods, olive oil, moderate poultry and fish intake, and restricting red meat, the Mediterranean diet has been associated with a decreased risk of a first stroke [18, 43]. Previous cohort studies (including the Ana cohort [44] and the Korean cohort [45) have shown that high DII scores are significant association between high DII scores and cardiovascular events, with several pieces of research supporting the direct association between DII and stroke [18, 20].Unlike the DII, which assesses overall dietary inflammatory potential based on 45 components, FISI integrates food-specific inflammation scores with daily NRVs, enabling more precise, individualized dietary recommendations. This distinction enhances FISI’s applicability in clinical and public health settings, allowing tailored interventions based on individual dietary patterns [46]. In addition, FII offers advantages in dietary guidance by providing food-specific scores, as illustrated in Fig. 7, which illustrates the shift from nutrient-focused to food-focused strategies for personalized advice.

### Pathophysiological mechanisms: the inflammatory cascade

One of the main pathological contributors to stroke is chronic low-grade inflammation [7, 8]. Our study reinforces that FISI’s ability to quantify dietary inflammatory potential offers a practical tool for mitigating these pathways. The biological mechanism follows a sophisticated logical chain: a pro-inflammatory diet triggers the systemic release of pro-inflammatory cytokines such as IL-6 and TNF-α, which elevates oxidative stress levels. This oxidative environment induces endothelial dysfunction, reducing nitric oxide bioavailability and impairing vascular reactivity [47]. Furthermore, persistent inflammation facilitates the oxidative modification of low-density lipoprotein (ox-LDL), accelerating foam cell formation and atherosclerotic plaque expansion. Simultaneously, elevated biomarkers like CRP, WBC, and NLR [8, 48–50] signal a pro-thrombotic state where the coagulation system is triggered, significantly increasing the risk of thrombosis and ischemic stroke [51].

### Clinical value of machine learning and SHAP interpretability

A major methodological strength of this study is the application of XGBoost coupled with SHAP analysis. Unlike traditional logistic regression, XGBoost can capture complex, non-linear connections and high-order interactions between diet and comorbidities. By employing SHAP analysis, we solved the “black box” problem of machine learning [52]. It allowed us to visualize exactly which dietary factors contribute most to an individual’s stroke risk, providing clinicians with a transparent and trustworthy tool for individualized risk prediction and precision nutrition interventions.

### Limitations

Despite its strengths, several limitations must be noted. First, the cross-sectional nature of this study precludes the establishment of temporal precedence or causal relationships. While our models show a strong association between high FISI and stroke prevalence, it remains possible that post-stroke lifestyle changes influenced dietary patterns. Future longitudinal studies or prospective cohorts are essential to validate these findings and elucidate the causal pathways between cumulative dietary inflammation and stroke incidence. Second, 24-hour dietary recall data might be subject to recall bias. Third, missing flavonoid data in NHANES may lead to an underestimation of the anti-inflammatory effects of certain foods. Fourth, although we used 10-fold cross-validation, the risk of overfitting in machine learning models cannot be entirely ruled out. Fifth, we were unable to account for the effects of medications like statins and antiplatelet drugs, which possess potent anti-inflammatory properties and could influence both inflammatory levels and stroke risk [53]. Finally, this study did not consider the gut microbiota—a key mediator between diet and systemic inflammation [54]. Future research should explore these factors to further validate FISI’s utility in diverse, high-stroke-burden populations [55, 56].

## Conclusions

This cross-sectional study indicates that elevated levels FISI34, FISI26-USDA, FISI26-CHINA are significantly associated with greater stroke prevalence among U.S. adults. Machine learning with Boruta and SHAP confirmed FISI34 as a key predictor of stroke. The FII, underpinning FISI, surpasses the DII by offering food-specific scores and expanded nutrient data for precise dietary assessment. These results emphasize dietary inflammation’s role in stroke and support FISI for personalized interventions. Additional studies are needed to determine causality and expand the use of FISI across different groups.

## Supplementary Information

Supplementary Material 1

## Data Availability

The datasets used and analyzed in this study are publicly available in the NHANES database ([https://www.cdc.gov/nchs/nhanes/](https:/www.google.com/url? sa=E&q=https%3 A%2 F%2Fwww.cdc.gov%2Fnchs%2Fnhanes%2 F)) *.* The raw data used for analysis can be requested from the first author of this article at [yanzhiwen@stu.cqmu.edu.cn](mailto: yanzhiwen@stu.cqmu.edu.cn).
