# Supplementary material for: Investigating the association between the food inflammation scores of individuals and stroke in adults: an extreme gradient boosting machine learning model interpreted with shapley additive explanations
Source: J Health Popul Nutr. 2026 May 18;45:170. doi: 10.1186/s41043-026-01342-6 (PMC13377719; doi:10.1186/s41043-026-01342-6)
Supplement: Supplementary file 1 — Supplementary Material 1 [file 41043_2026_1342_MOESM1_ESM.pdf]

Supplementary Materials

The following supplementary materials provide additional details supporting the main text of the study.

Supplementary Information S1. Calculation of FISI34, FISI26-USDA, FISI26- CHINA and DII

Supplementary Table S1. The total inflammation score (TIS) and nutrient recommended values (NRVs) of nutrient components for Food Inflammation Score of Individuals (FISI) Calculations

Supplementary Table S2. Food Parameters, Inflammatory Effect Scores, and Global Intake Values for Dietary Inflammatory Index (DII)

Supplementary Table S3. Characteristics of Study Participants Before and After Propensity Score Matching

Supplementary Table S4. Multivariable Logistic Regression Analysis of the Association Between Dietary Inflammatory Indices and Stroke After Propensity Score Matching

Supplementary Information S1. Calculation of FISI34, FISI26-USDA, FISI26- CHINA and DII

Overview

The **information** outlines the detailed methodology for calculating the Food Inflammation Score of Individuals (FISI) variants (FISI34, FISI26-USDA, FISI26-CHINA) and the Dietary Inflammatory Index (DII) based on dietary intake data from the National Health and Nutrition Examination Survey (NHANES).

FISI34

The FISI34 used in this study is derived from the FISI39 framework, which is based on 39 key nutrients as outlined in Wang et al. (2024)[1]. However, due to the NHANES 2011-2018 data cycles lacking data on Flavan-3-ols, Flavanones, Flavones, Flavonols, and Isoflavones, the nutrient count was adjusted to 34.

FISI34 includes 34 dietary components, with NRVs based on the U.S. Dietary Guidelines for Americans (DGA) 2020-2025 for individuals aged 31-50 at a 2200 kcal level[2]. The components are: Protein, Total lipid (fat), Carbohydrate, Energy, Alcohol, Caffeine, Fiber, Iron, Magnesium, Zinc, Selenium, Vitamin A (RAE), Alpha-carotene, Vitamin E (alpha-tocopherol), Vitamin D (D2 + D3), Vitamin C, Thiamin, Riboflavin, Niacin, Vitamin B-6, Vitamin B-12, Folic acid, Added Vitamin E, Added Vitamin B-12, Cholesterol, Saturated fatty acids, PUFA 18:2, PUFA 18:3, PUFA 20:4, PUFA 22:6 n-3 (DHA), PUFA 20:5 n-3 (EPA), PUFA 22:5 n-3 (DPA), Total monounsaturated fatty acids, Total polyunsaturated fatty acids.

FISI26-USDA

FISI26-USDA excludes eight components from FISI34: Alcohol, Caffeine, Vitamin D, Vitamin B-6, Vitamin B-12, Folic acid, Added Vitamin E, and Added Vitamin B-12. This leaves 26 major dietary components, with the same calculation method and NRVs as FISI34, based on DGA 2020-2025.

FISI26-CHINA

FISI26-CHINA uses the same 26 components as FISI26-USDA but adjusts NRVs according to the Dietary Reference Intakes for China 2023 Edition for men aged 50[3].

DII Calculation Methodology

The DII calculation follows the standardized methodology outlined in Shivappa et al. (2014) [4]and implemented using the R package dietaryindex (*https://github.com/jamesjiadazhan/dietaryindex*)[5]. The 28 components included are: Carbohydrates, Protein, Alcohol, Fiber, Cholesterol, Total fat, Saturated fat, Monounsaturated fatty acids (MUFA), Polyunsaturated fatty acids (PUFA), n-3 fatty acids, n-6 fatty acids, Vitamins A, B1, B2, B3, B6, B12, C, D, E, Iron, Magnesium, Zinc, Selenium, Folic acid, Beta-carotene, Caffeine, and Energy. The DII scoring algorithm involves:

1. Calculating z-scores for each dietary component by subtracting the global mean and dividing by the global standard deviation, based on a reference global database [4].
2. Converting z-scores to centered percentiles to normalize the distribution.
3. Multiplying each centered percentile by the component's inflammatory effect score (provided in Supplementary Table S2) to obtain component-specific DII scores.
4. Summing all component-specific DII scores to derive the overall DII score for an individual. Inflammatory effect scores for the 28 components are provided in Supplementary Table S2.

Supplementary Table S1. The total inflammation score (TIS) and nutrient recommended values (NRVs) of nutrient components for Food Inflammation Score of Individuals (FISI) Calculations

Name	Unit name	TIS	NRVs	NRVs (China)
Protein	G	0.021	56	65
Total lipid (fat)	G	0.298	77	54.17
Carbohydrate, by difference	G	0.097	130	120
Energy	KCAL	0.18	2200	1950
Fiber, total dietary	G	-0.663	31	30
Iron, Fe	MG	0.032	8	12
Magnesium, Mg	MG	-0.484	420	320
Zinc, Zn	MG	-0.313	11	12
Selenium, Se	UG	-0.191	67	60
Vitamin A, RAE	UG	-0.401	900	750
Carotene, alpha	UG	-0.584	3718	3718
Vitamin E (alpha-tocopherol)	MG	-0.419	15	14
Vitamin C, total ascorbic acid	MG	-0.424	90	100
Thiamin	MG	-0.098	1.2	1.4
Riboflavin	MG	-0.068	1.3	1.4
Niacin	MG	-0.2462	16	15
Cholesterol	MG	0.11	279.4	279.4
Fatty acids, total saturated	G	0.373	22	21.67
PUFA 18:2	G	-0.159	10.8	12.46
PUFA 18:3	G	-0.436	1.06	2.71
PUFA 20:4	G	-0.159	10.8	12.46
PUFA 22:6 n-3 (DHA)	G	-0.436	1.06	2.71
PUFA 20:5 n-3 (EPA)	G	-0.436	1.06	2.71
PUFA 22:5 n-3 (DPA)	G	-0.436	1.06	2.71
Fatty acids, total monounsaturated	G	-0.009	27	27
Fatty acids, total polyunsaturated	G	-0.337	13.88	15.17
Alcohol, ethyl	G	-0.278	5.15	
Caffeine	MG	-0.11	8.05	
Vitamin D (D2 + D3)	UG	-0.446	15	
Vitamin B-6	MG	-0.365	1.3	
Vitamin B-12	UG	0.106	2.4	
Folic acid	UG	-0.19	400	
Vitamin E, added	MG	-0.419	15	
Vitamin B-12, added	UG	0.106	2.4	
Flavan-3-ols	mg	-0.415	95.8	
Flavanones	mg	-0.25	11.7	
Flavones	mg	-0.616	1.55	
Flavonols	mg	-0.467	17.7	
Isoflavones	mg	-0.593	1.2	

The total inflammation score (TIS) and nutrient recommended values (NRVs) of nutrient components. NRVs referred to the age of 31-50 and calorie level at 2200 kcal in the DGA 2020-2025. NRVs (China) referred to men aged 50 in the Dietary Reference Intakes for China 2023[1].

Supplementary Table S2. Food Parameters, Inflammatory Effect Scores, and Global Intake Values for Dietary Inflammatory Index (DII)

Food Parameter	Raw Inflammatory Effect Score*	Overall Inflammatory Effect Score†	Global Daily Mean Intake‡ (units/d)	sd‡
Protein (g)	0.049	0.021	79.4	13.9
Total fat (g)	0.298	0.298	71.4	19.4
Carbohydrate (g)	0.109	0.097	272.2	40
Energy (kcal)	0.18	0.18	2056	338
Fibre (g)	-0.663	-0.663	18.8	4.9
Iron, Fe (mg)	0.032	0.032	13.35	3.71
Magnesium, Mg (mg)	-0.484	-0.484	310.1	139.4
Zinc, Zn (mg)	-0.313	-0.313	9.84	2.19
Selenium, Se (μg)	-0.191	-0.191	67	25.1
Vitamin A (RE)	-0.401	-0.401	983.9	518.6
Vitamin E (mg)	-0.419	-0.419	8.73	1.49
Vitamin C (mg)	-0.424	-0.424	118.2	43.46
Thiamin (mg)	-0.354	-0.098	1.7	0.66
Riboflavin (mg)	-0.727	-0.068	1.7	0.79
Niacin (mg)	-1	-0.246	25.9	11.77
Cholesterol (mg)	0.347	0.11	279.4	51.2
Saturated fat (g)	0.429	0.373	28.6	8
MUFA (g)	-0.019	-0.009	27	6.1
PUFA (g)	-0.337	-0.337	13.88	3.76
n-3 Fatty acids (g)	-0.436	-0.436	1.06	1.06
n-6 Fatty acids (g)	-0.159	-0.159	10.8	7.5
Vitamin B6 (mg)	-0.379	-0.365	1.47	0.74
Vitamin B12 (μg)	0.205	0.106	5.15	2.7
Folic acid (μg)	-0.207	-0.19	273	70.7
Vitamin D (μg)	-0.446	-0.446	6.26	2.21
Caffeine (g)	-0.124	-0.11	8.05	6.67
Alcohol (g)	-0.278	-0.278	13.98	3.72
β-Carotene (μg)	-0.584	-0.584	3718	1720

RE, retinol equivalents; *: The value of Raw Inflammatory Effect Score, overall inflammatory effect score, global daily mean intake (units/d) and sd are derived from references(Shivappa N, Steck SE, Hurley TG, Hussey JR, Hebert JR. Designing and developing a literature-derived, population-based dietary inflammatory index. Public Health Nutr. 2014;17:1689-96.).[4]

Supplementary Table S3. Characteristics of Study Participants Before and After Propensity Score Matching

	Unmatched^			Matched^		
**Characteristic**	**Non-stroke** N = 18,883[1]	**Stroke** N = 798[1]	**p-value**[2]	**Non-stroke** N = 798[1]	**Stroke** N = 798[1]	**p-value**[2]
**Gender**			0.630			0.291
Female	10,162 (54%)	422 (53%)		444 (56%)	422 (53%)	
Male	8,721 (46%)	376 (47%)		354 (44%)	376 (47%)	
**Age**	50.1 ± 17.5	66.4 ± 12.5	**< 0.001**	66.4 ± 12.7	66.4 ± 12.5	0.956
**Race**			**< 0.001**			0.757
Mexican American	2,757 (15%)	62 (7.8%)		56 (7.0%)	62 (7.8%)	
Non-Hispanic Black	3,799 (20%)	213 (27%)		220 (28%)	213 (27%)	
Non-Hispanic White	8,366 (44%)	421 (53%)		409 (51%)	421 (53%)	
Other Hispanic	1,919 (10%)	50 (6.3%)		62 (7.8%)	50 (6.3%)	
Other Race	2,042 (11%)	52 (6.5%)		51 (6.4%)	52 (6.5%)	
**education level**			**< 0.001**			0.900
< High school	1,603 (8.5%)	105 (13%)		105 (13%)	105 (13%)	
> High school	14,874 (79%)	549 (69%)		542 (68%)	549 (69%)	
Completed high school	2,406 (13%)	144 (18%)		151 (19%)	144 (18%)	
**Marital status**			**< 0.001**			0.960
Married/Living with partner	11,492 (61%)	426 (53%)		424 (53%)	426 (53%)	
Widowed/Divorced /Separated/Never married	7,391 (39%)	372 (47%)		374 (47%)	372 (47%)	
**Alcohol consumption**			**< 0.001**			0.453
Current heavy drinkers	5,605 (30%)	115 (14%)		108 (14%)	115 (14%)	
Current moderate drinkers	5,847 (31%)	207 (26%)		235 (29%)	207 (26%)	
Former drinkers	4,765 (25%)	363 (45%)		352 (44%)	363 (45%)	
Lifetime abstainers	2,666 (14%)	113 (14%)		103 (13%)	113 (14%)	
**Smoke status**			**< 0.001**			0.889
Current smoker	3,291 (17%)	181 (23%)		173 (22%)	181 (23%)	
Former smoker	4,668 (25%)	304 (38%)		309 (39%)	304 (38%)	
Never smoker	10,924 (58%)	313 (39%)		316 (40%)	313 (39%)	
**BMI**	29.5 ± 7.1	30.3 ± 7.0	**0.003**	30.1 ± 6.9	30.3 ± 7.0	0.653

Characteristic	Unmatched^ Non-stroke N = 18,883[1]	Stroke N = 798[1]	p-value[2]	Matched^ Non-stroke N = 798[1]	Stroke N = 798[1]	p-value[2]
**Hypertension**	8,047 (43%)	651 (82%)	**< 0.001**	653 (82%)	651 (82%)	0.948
**CKD**	3,354 (18%)	374 (47%)	**< 0.001**	384 (48%)	374 (47%)	0.652
**Hyperlipidemia**	13,672 (72%)	701 (88%)	**< 0.001**	714 (89%)	701 (88%)	0.343
**diabetes**	3,597 (19%)	344 (43%)	**< 0.001**	344 (43%)	344 (43%)	1.000

[1] 1Welch Two Sample t-test; Pearson's Chi-squared test
[2] Pearson's Chi-squared test; Welch Two Sample t-test

Supplementary Table S4. Multivariable Logistic Regression Analysis of the Association Between Dietary Inflammatory Indices and Stroke After Propensity Score Matching

**Model 3**	**OR**	**95% CI**	**p-value**
FISI26-CHINA	1.11	1.00, 1.24	0.050
FISI26-USDA	1.10	1.00, 1.20	0.048
FISI34	1.06	1.02, 1.11	**0.006**
DII	1.08	1.01, 1.16	0.018

Abbreviations: CI = Confidence Interval, OR = Odds Ratio. Adjusted for age, gender, race, marital status, education level, poverty-to-income ratio (PIR), body mass index (BMI), smoking status, alcohol consumption, hypertension, diabetes, CVD, CKD, cancer.

References

1. Wang Z, Yuan C, Zhang Y, Abdelaty NS, Chen C, Shen J, Zhang L, Lu B, Liu R, Li P: **Food inflammation index reveals the key inflammatory components in foods and heterogeneity within food groups: How do we choose food?** *Journal of advanced research* 2024.
2. U.S. Department of Agriculture, & U.S. Department of Health and Human Services. (2020). **Dietary Guidelines for Americans**, 2020-2025 (9th ed.). U.S. Government Publishing Office. https://www.dietaryguidelines.gov/sites/default/files/2020-12/Dietary_Guidelines_for_Americans_2020-2025.pdf
3. YX Y: **Society CN,Dietary Reference Intakes for China**. Beijing: People's Medical Publishing House; 2023.
4. Shivappa N, Steck SE, Hurley TG, Hussey JR, Hébert JR: **Designing and developing a literature-derived, population-based dietary inflammatory index**. *Public health nutrition* 2014, **17**(8):1689-1696.
5. Zhan JJ, Hodge RA, Dunlop AL, Lee MM, Bui L, Liang D, Ferranti EP: **Dietaryindex: a user-friendly and versatile R package for standardizing dietary pattern analysis in epidemiological and clinical studies**. *The American journal of clinical nutrition* 2024, **120**(5):1165-1174.
